# Novel MLPA procedure using self-designed probes allows comprehensive analysis for CNVs of the genes involved in Hirschsprung disease

**DOI:** 10.1186/1471-2350-11-71

**Published:** 2010-05-11

**Authors:** Avencia Sánchez-Mejías, Rocio Núñez-Torres, Raquel M Fernández, Guillermo Antiñolo, Salud Borrego

**Affiliations:** 1 Unidad de Gestión Clínica de Genética, Reproducción y Medicina Fetal. Instituto de Biomedicina de Sevilla (IBIS), Hospitales Universitarios Virgen del Rocío/CSIC/Universidad de Sevilla, Sevilla, Spain; 2 Centro de Investigación Biomédica en Red de Enfermedades Raras (CIBERER), Sevilla, Spain

## Abstract

**Background:**

Hirschsprung disease is characterized by the absence of intramural ganglion cells in the enteric plexuses, due to a fail during enteric nervous system formation. Hirschsprung has a complex genetic aetiology and mutations in several genes have been related to the disease. There is a clear predominance of missense/nonsense mutations in these genes whereas copy number variations (CNVs) have been seldom described, probably due to the limitations of conventional techniques usually employed for mutational analysis. In this study, we have looked for CNVs in some of the genes related to Hirschsprung (*EDNRB, GFRA1, NRTN *and *PHOX2B*) using the Multiple Ligation-dependent Probe Amplification (MLPA) approach.

**Methods:**

CNVs screening was performed in 208 HSCR patients using a self-designed set of MLPA probes, covering the coding region of those genes.

**Results:**

A deletion comprising the first 4 exons in *GFRA1 *gene was detected in 2 sporadic HSCR patients and *in silico *approaches have shown that the critical translation initiation signal in the mutant gene was abolished. In this study, we have been able to validate the reliability of this technique for CNVs screening in HSCR.

**Conclusions:**

The implemented MLPA based technique presented here allows CNV analysis of genes involved in HSCR that have not been not previously evaluated. Our results indicate that CNVs could be implicated in the pathogenesis of HSCR, although they seem to be an uncommon molecular cause of HSCR.

## Background

Hirschsprung disease (HSCR, OMIM 142623) is a congenital malformation characterized by the absence of intramural ganglion cells in the myenteric and submucosal plexuses along a variable portion of the distal intestine, due to a defect of craniocaudal migration of neuroblasts originated from the neural crest [1,2]. HSCR presents an estimated incidence of 1/5000 live births, and has a non mendelian inheritance with reduced penetrance, variable expression and male predominance. Although familial forms exist, the vast majority of cases are sporadic. In addition, the disease can present as an isolated trait, although in a 30% of the cases it is associated with chromosomal abnormalities, neurodevelopment disorders and a variety of additional isolated anomalies and syndromes [2].

HSCR has a complex genetic aetiology with several genes being described as associated with isolated or syndromic forms. *RET *proto-oncogene is considered the major causal gene in HSCR and has been extensively studied in different HSCR series worldwide. Both traditional *RET *coding mutations and a common non-coding *RET *variant within a conserved enhancer-like sequence in intron 1, have been reported to be associated with a great proportion of HSCR cases [2-4]. Other genes associated with HSCR encode for receptors, ligands (especially those participating in the *RET *and *EDNRB *signaling transduction pathways), and transcriptional factors, such as *SOX10 *and *PHOX2B*, among others, that are usually involved in the neural crest cell development and migration [2].

Interestingly, many recent reports point out the implications of altered gene dosage in diagnosis, prognosis and therapy in different human diseases [5]. Nonetheless, it does not seem to be apparently the case of HSCR, with the current data supporting a predominance of missense/nonsense mutations, although small deletions/insertions have been occasionally observed (Human Gene Mutation Database of the Institute of Medical Genetics in Cardiff, http://www.hgmd.cf.ac.uk/ac/index.php). In fact, no duplications and only one gross deletion affecting the entire sequence of *RET *have been reported [6,7]. To date, only 2 studies have been reported investigating gene dosages anomalies in HSCR patients based on MLPA technique (Multiple Ligation-dependent Probe Amplification) [8,9], which has an optimal performance to detect alterations of gene dosages [10]. Both of them used MLPA MRC-Holland commercial kit for HSCR, that analyses a limited number of genes (*RET, ZEB2, EDN3 *and *GDNF*), and revealed no CNVs associated to HSCR in those genes [8,9]. In additions we have performed a *SOX10 *deletion screening on our HSCR patients [11] based on a previously reported QMF-PCR method (Quantitative Multiplex Fluorescent PCR), obtaining negative results [12]. Nevertheless, studies in other "HSCR genes" are necessary to rule out the potential implication CNVs in the pathogenesis of HSCR.

In the present study we have analyzed the presence of CNVs for *EDNRB, NRTN, GFRA1 and PHOX2B *in our patient series, using self-designed MLPA probes, as no commercial kit is available for those genes, and none of them has been previously evaluated for mid-size deletions/duplications using a high-throughput technique. The present self-design set of probes for MLPA analysis, together with the available MLPA commercial kit for HSCR, would lead to the complete analysis of CNVs within coding region of the most prevalent genes in HSCR.

## Methods

### Patients and Control Subjects

In this study, a total of 208 HSCR patients have been included (22% female, 77% male). 188 out of the 208 patients were sporadic cases, while 20 were familial cases belonging to 13 different families. In addition, 6 of those patients presented with associated Down's syndrome, and 1 presented with Waarbenburg's Syndrome type 4. In order to define the exact HSCR phenotype in our patients, we have used the criteria recommended by Chakravarti and Lyonnett [1]. Following these criteria, 137 cases were catalogued as short-segment HSCR (S-HSCR, 81%), 21 cases as long-segment (L-HSCR, 12%), and 12 cases presented as total colonic aganglionosis (TCA, 7%). Data were not available for the remaining 38 cases.

We have also used a group of 100 controls comprising unselected, unrelated, race, age, and sex-matched individuals. All of them were healthy voluntary donors, who came to the Hospital for other reasons and did not present any symptom suggestive of HSCR.

Genomic DNA was extracted according to standard protocols and an informed consent was obtained from all the participants for clinical and molecular genetic studies. The study conformed to the tenets of the declaration of Helsinki and was approved by the Hospitales Universitarios Virgen del Rocío IRB.

### MLPA analysis

Gene dosage variations on *EDNRB, GFRA1, NRTN *and *PHOX2B *were analysed by MLPA technology. The selection of the genes was based on their implication in ENS development and HSCR disease [13]. More specifically, we have selected *EDNRB*, the second major gene for HSCR, which has a considerably higher mutational incidence than *EDN3*, *GDNF *or *ZEB2 *[2], included in the commercial kit. Since *RET *and *GDNF *are already included in the commercial MLPA kit for HSCR, we decided to include *NRTN *and *GFRA1*, as they are implicated in the same signaling pathway and have been previously associated to HSCR [14,15]. In addition and due to the implication of *PHOX2B *deletions in human pathologies and syndromes than frequently present with HSCR (CCHS) [16], we have also included this gene in the present study. Following MRC-Holland recommendations, we designed 31 sets of probes to detect deletions and duplications in one or more exons of these 4 genes (Table 1). In addition, we designed 3 control fragments hybridizing to different genome regions, that have never been associated with HSCR before and have been reported to contain no CNVs. Probes and EK-1 kits were supplied by Sigma (Sigma-Aldrich, St. Louise, MO) and MRC-Holland (MRC-Holland, Amsterdam, Netherlands) respectively.

**Table 1:** Self-designed MLPA probes used in the molecular analysis of *EDNRB, GFRa1, NRTN *and *PHOX2B *CNVs of 208 HSCR patients.

	Exon	Probe Oligo Sequence*	bp
EDNRB	EDNRBen1Δ3	LPO TCTGGCGGTGATTGATGGGAAG	100
		RPO GGATGAATGAATAAAAGTACTTGTCTGATGGCACCC	
	EDNRBex1	LPO TCTACAAGAACAAGTGCATGCGAAACG	112
		RPO GTCCCAATATCTTGATCGCCAGCTTGCCATCAATCGCCATTCGA	
	EDNRBex2	LPO GGCAGAGGACTGGCCATTTGGAG	90
		RPO CTGAGATGTGTAAGCTGGTGCCTTT	
	EDNRBex3	LPO CGACAGCAGTAGAAATTGTTTTGATTTGGGTG	128
		RPO GTCTCTGTGGTTCTGGCTGTCCCTGAAGAGGTTTTGTGTACGGACCTAAAGTTC	
	EDNRBex4	LPO TGGCATGCAGATTGCTTTAAATGATCAC	113
		RPO CTAAAGCAGGTAAGAAAATACAAATATTGAGAGGGACACGGCG	
	EDNRBex5	LPO GTGGCCAAAACCGTCTTTTGCCTG	93
		RPO GTCCTTGTCTTTGCCCTCTGCTGGCTT	
	EDNRBex6	LPO CGATGCTATTCACATAACCCAATTGCTCTGTATTTGGTGAG	130
		RPO GTGAGCAAAAGATTCAAAAACTGCTCTTGGAGGAAGTCGAGGAGTAC	
	EDNRBex7	LPO GCAGTCGTGCTTAAAGTTCAAAG	99
		RPO CTAATGATCACGGATATGACAACTTTGCTGAGTG	

GFRα1	GFRa1ex1	LPO CCTAGCGCAGATAAAGTGAGCCCGGAAAG	135
		RPO GGAAGGAGGGGGCGGGGACACCATTGCTATAGACGTAGCTGTGAGTACCAACCGAATAGCAATC	
	GFRa1ex2	LPO CAACGACTAGAGAGGCACCATGTTCCTGGCGACC	127
		RPO GATGGAGCTGAACTTTGGGCGGCCAGTGACTGCCTTGAAGGTCTCACGGCT	
	GFRa1ex3	LPO GGAGAAGAACTGCCTGCGCATTTACTGGAG	96
		RPO CATGTACCAGAGCCTGCAGGGTAC	
	GFRa1ex4	LPO GGAGGATTCCCCATATGAACCAGTTAACAG	100
		RPO CAGATTGTCAGATATATTCCGGGTGGTC	
	GFRa1ex5	LPO CAGGCACTTGAGGATTTCCCAGGTAGGACCCTCTAGTTGCAG	131
		RPO GAAAACAAGGTCAGGGCTGCCACTGGTTCTATAATACAATGGAGACG	
	GFRa1ex6	LPO GGAACAACTGCCTGGATGCAGCGAAGGCCTG	108
		RPO CAACCTCGACGACATTTGCAAGAAGTACCCGGTAT	
	GFRa1ex7	LPO TGCCAGCCAGAGTCAAGGTCTGTCAG	95
		RPO CAGCTGTCTAAAGGAAAACTACGCTGA	
	GFRa1ex8	LPO CCATACATCACCGCATTCTCGCAGAAGAGCCTCAGTGTGGCCCCATGGTGTGACTG	132
		RPO CAGCAACAGTGGGAACGACCTAGAAGAGTGCTTG	
	GFRa1ex9a	LPO GAGGAAGTTCAATGGCTCCGATGTGACCGTGTGGCAG	108
		RPO CCAGCCTTCCCAGTACAGACCACCACTGC	
	GFRa1ex9b	LPO CCAATTGACCACCTATGGGCCCAGTCC	140
		RPO CCAGTCAGGTCAAAGAAGAGGGTTTACGTAGACCTCAGTTCACTGGAGTCTGTCGTTTGCTCTATACTCAC	
	GFRa1ex9c	LPO GGTTCAGGCAACACAGAGACAAAG	91
		RPO CATCTTCAGGGGGAGCAGGTAGAGG	
	GFRa1ex9d	LPO GTGTCCACTTGTTTTACGCAG	108
		RPO CTGACTTTACTGGACATTATTCAGACCAGTGGTTGGGTGCGCTCG	
	GFRa1ex10	LPO GCTGAAATCCAATGTGTCGGGCAATACACAC	112
		RPO CTCTGTATTTCCAATGTAAGTATGGGCGTGGACTCGCAT	
	GFRa1ex11	LPO CCAAACTGTGCGCGTGTCGATAAGGTTCCGTTCCCGCCCACTGCTGGTCCTGGTGGTAACC	136
		RPO GCTCTGTCCACCCTATTATCTTTAACAGAAACA	

NRTN	NRTNex1	LPO CGTTCAAAGTCAAAGGCCCCACACTGAGTC	136
		RPO CTGGCCCAGCGCCCTGTGCCCGTTGGCTGACGAGAAGTACGGAATCGAATCTATAGTGACCTAT	
	NRTNex2	LPO CGAATTAGAGATTTAACTTCCTCCCCTCGCAGACCGTGCACTC	139
		RPO CTGCAGGGGGCCCCGGATGCGATGGAGCTGTTAATACGACTCACTATAGGAGTA	

PHOX2B	PHOX2B5'UTR	LPO CTTAAATCATGGGGCCACTGAAGTC	92
		RPO CACACACTGCTCGCTCCTTTGT	
	PHOX2Bex1A	LPO CCTCAATTCCTCTGCCTACGAGTC	88
		RPO CTGTATGGCTGGGATGGACACC	
	PHOX2Bex1B	LPO CCAGTGGCTTCCAGTATAACCCGATAAGGAC	96
		RPO CACTTTTGGGGCCACGTCCGGCT	
	PHOX2Bex2	LPO CTGTCATACTCTAGTTCCTTACAAACTCTTCAC	108
		RPO GGACCACGGCGGCCTCAACGATGCAAGCGACAT	
	PHOX2Bex3A	LPO TCTTCTTTCTCCCCCTGCTTCACCGTCTCTC	112
		RPO CTTCCGTCTTGGGCCAGGTGTGGTTCCAGAATTTACTAA	
	PHOX2Bex3B	LPO CGTCCTATCTTCGCTCCAAAGACCCAACG	100
		RPO GTGCCAAAGCCGCCTTAGTGAAGAGCAGT	
	PHOX2B3'UTR	LPO CTTTTTCATTGAGGGCCTAAAGTAATCGCGCTAAGAATAAAG	126
		RPO GGAAAACGGCGTCGCCCTCATTTGCAAACTGTGCGGGTGTCG	

Reference genes	TOR1A	LPO GCACCGGCAAAAATTTCGTCAGCAAGATCAT	104
		RPO CGCAGAGAATATTTACGAGGGTGGTCTGAAC	
	EPO	LPO GCCTCAGCTGCTCCACTCCGAACAATCACTG	117
		RPO CTGACACTTTCCGCAAACTCTTCCGAGTCTAGATAGTTCCAACAA	
	SS18	LPO CGACAGCATTACGAAGCACAGCAGCCACCTATGGGAATGATG	122
		RPO GGTCAAGTTAACCAAGGCAATCATATGATGCGATATGC	

*Sequences do not included the universal primers located al the 5' end of LPO and 3' end of RPO.

Capillary electrophoresis analysis was performed using an ABI PRISM^® ^3730 DNA analyzer (Applied Biosystems, Foster City, CA) and for data analysis we used GeneMarker v 1.75 (Softgenetics L.L.C, State College, PA). We normalized the samples by peak height comparing patients with 10 controls. These 10 control individuals had been confirmed to have no duplications or deletions in the studied genes, by a previous analysis using Affymetrix Genome-Wide Human SNP Arrays 6.0. In addition as a positive control we included a patient harbouring a *GFRA1 *deletion in heterozygosis (patient HSCR-5, presenting with TCA-total colonic aganglionosis), which had been previously characterized by southern blot and lost of heterozygosity of STRs [15]. This individual not only was useful as positive control, but also confirmed the validity of our method to detect deletions in the genes analysed. Following manufacturer recommendations, dosage quotients under 0.5 or over 1.3 were considered as indicating potential deletions or duplications respectively, and were confirmed in 3 independent assays.

## Results

With the aim to analyse anomalies in the gene dosage of several genes described as associated to HSCR (*EDNRB, GFRA1, NRTN *and *PHOX2B*), but never previously analyzed by MLPA, we designed specific synthetic MLPA D-probes, following MRC-Holland recommendations. The hybridization, ligation and amplification of the MLPA probes were performed in 4 different probemixes of 8-10 probes each, together with the 3 control probes. Signal peaks height of the amplified products observed after electrophoresis, were as homogeneous as expected for self-designed probes, and peak normalization was successfully fulfilled between the patient samples and controls in all the probemixes tested.

**Figure 1:**
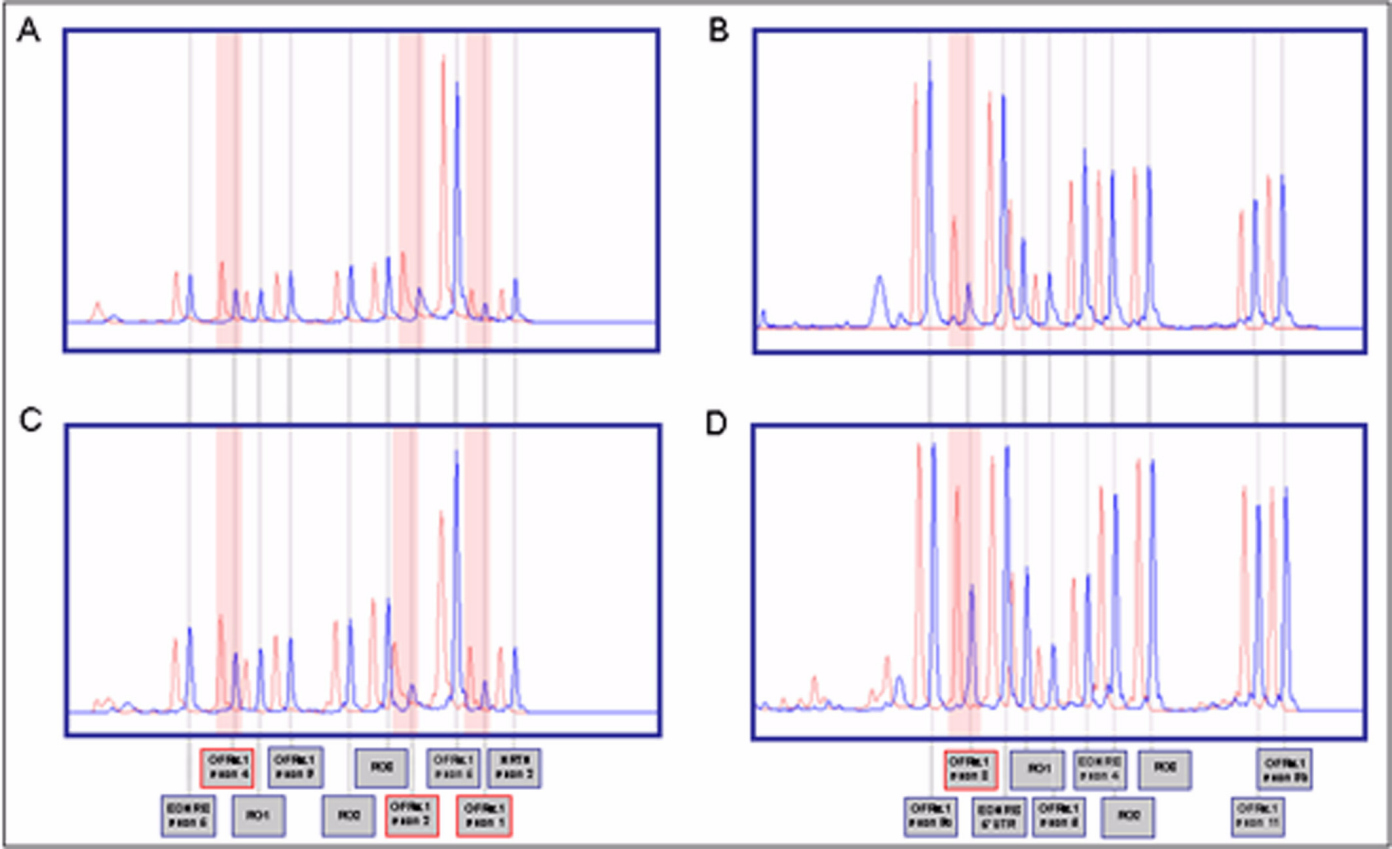
**MLPA profiles for control individuals (red) and for the HSCR patient (blue)**. Two different probes-mixes are shown (A and C mix 1; B and D mix 2). For those HSCR patients (A and B, HSCR-115; C and D, HSCR-5), it was observed a decrease for the dosage of *GFRA1 *exons 1, 2, 3 and 4, highlighted in red boxes and by arrows.

After the validation of our probes, we screened a total of 208 HSCR patients and found a deletion in *GFRA1 *gene (c.(?-555)_431+?del; Figure 1) that affects exons 1a, 2a, 3 and 4 in isoform NM_005264 and exons 2b, 3 and 4 in isoform NM_145793. This deletion was detected in a heterozygous state, in a sporadic and isolated male HSCR patient presenting with short-segment HSCR (patient HSCR-115), and was not found in 100 control individuals tested. This deletion was inherited by his unaffected father, and was found to be absent in other healthy members of the family. There are 2 CNVs described for *GFRA1 *gene annotated in the Database of Genomics Variants http://projects.tcag.ca, Variant_48418 and Variant_48004. The first variant is a 2.5Kb deletion located in the 3' untranslated region of the gene, while the other consisted on a 36 Kb deletion the genomic region containing exons 7, 8 and 9 of *GFRA1*. Therefore, the available data support that we are describing a novel deletion. Interestingly, this is the second time this deletion has been found in our HSCR patient series, in a patient not related with the one previously reported [Figure 1; 15].

In order to preliminarily examine the potential damaging effect of this deletion on *GFRA1 *expression and functionality, we used InterProScan and AlternativeSplicing tools from EBI and Transec from EMBOSS. We verified that the critical translation initiation signal in the gene was abolished; subsequently no wild-type (WT) protein was expected to be expressed from the deleted copy of the gene. In adition, we checked *in silico *whether the deleted allele could produce any protein with similar functional capacity as GFRα1. We found that an alternative peptide could be translated from deleted isoform NM_005264 with the same carboxyl-terminus aminoacidic sequence. This putative protein would maintain one of the 3 GDNF/GAS1 domains in the WT protein, but would also lack the localization signal in the N-terminal region. Although this deletion is well refined in its 3' end, we failed to establish the boundaries in the 5' end where all transcription and translation signals are located. Therefore it seems unlikely that the aberrant protein could be expressed, and in that case it would have a very limited, or even null, functionality.

## Discussion

HSCR has a complex genetic aetiology and point mutations in several genes have been reported to be implicated in a portion of isolated and syndromic HSCR forms [2]. It is tempting to speculate that other genetic events different from point mutation, such as CNVs, have a functional role in the pathogenesis of HSCR. Very little is known in this field for HSCR since typical screening methods based in conventional PCR are only able to detect small deletions/duplications (a few base pairs), and cytogenetic techniques can exclusively detect alterations in the order of megabases. Those techniques are neither powerful nor adequate to detect CNVs [10], so that those types of rearrangements would be missed. In this way, it would be possible that such mid-size deletions/duplications in several HSCR genes have been underreported. In addition, traditional techniques used to detect mid-size deletions/duplications, such as southern blot, are expensive, time consuming and not suitable for high-throughput results. For this reason we planned to perform CNVs screening in a large series of HSCR patients using MLPA technology, which can be performed in a large number of individuals within a short period time, in order to determine if it is a reliable technique suitable for a routine CNVs screening. Despite the negative results previously reported for HSCR MLPA commercial kit [8,9], we have obtained positive results with the finding of a deletion affecting the 4 first exons in *GFRA1*. This deletion was previously identified in a sporadic HSCR patient, but its actual implication in the pathogenesis of this disease remained unknown [15]. The finding of the same deletion in an independent patient with the same phenotype and its absence in the control population, support that this deletion at the *GFRA1 locus *is a mutational event potentially related to HSCR. In addition, the implementation of MLPA technique for midsize deletion detection leads us to refine the deleted region at *GFRA1 *locus. The protein GFRα1 is one of the four co-receptors of the RET tyrosine kinase receptor. The binding of RET to GFRα1 is required for the specific recruitment of GDNF and the subsequent phosphorylation of RET. Therefore, the presence of such a deletion in GFRα1 would avoid the expression of the protein, presumably preventing RET phosphorylation and affecting the correct development of the ENS. The presence of this mutation in unaffected members of the family suggest that it could be necessary but not sufficient to produce the phenotype, and additional unidentified genetic events might be acting in this HSCR patient. In this sense, no point coding mutations were detected in this patient, or in the previously described patient harbouring the same deletion, in other HSCR-related genes tested such as *RET*, *GDNF, NRTN, PSPN, ARTN, EDNRB*, *EDN3*, *NTF3, NTRK3, SOX10 *or *PHOX2B*. The present results indicate that CNVs are not a common molecular cause of HSCR, although they should be taken into account for further studies.

## Conclutions

One of our goals was to provide a simple, reliable, economic and fast method for CNVs screening in HSCR related genes, and the present study has successfully validated the self-designed MPLA probes for CNVs analysis. The design and validation of MLPA probes for additional genes represent an implementation for a technique that was restricted to the commercial production. In this sense, the present design, together with the commercial MLPA kit for HSCR, allows the complete analysis of CNVs in the coding region of the most prevalent genes for HSCR. In addition, the presence of a *GFRA1 *deletion that seems to impair protein function, in an unrelated HSCR patient supports and confirms the idea that this specific deletion might participate in the development of HSCR. Despite the fact that CNVs seems to be an uncommon susceptibility factor leading to this disease, our results point out the importance of taking into account those molecular events in HSCR studies from now on, at least in *GFRA1 *gene. Further screening of CNVs in additional series of patients would be necessary in order to completely address its real implications in the pathogenesis of HSCR.

## Competing interests

The authors declare that they have no competing interests.

## Authors' contributions

AS-M and RN-T carried out the molecular genetic studies and participated in the MLPA analysis. AS-M and SB participated in the design of the study and drafted the manuscript. RMF and GA helped to draft the manuscript. All authors have read and approved the final manuscript.

## Pre-publication history

The pre-publication history for this paper can be accessed here:

http://www.biomedcentral.com/1471-2350/11/71/prepub
